# Importin alpha binding and nuclear localization of PARP-2 is dependent on lysine 36, which is located within a predicted classical NLS

**DOI:** 10.1186/1471-2121-9-39

**Published:** 2008-07-21

**Authors:** Sandra S Haenni, Matthias Altmeyer, Paul O Hassa, Taras Valovka, Monika Fey, Michael O Hottiger

**Affiliations:** 1 Institute of Veterinary Biochemistry and Molecular Biology, University of Zurich, Winterthurerstrasse 190, 8057 Zurich, Switzerland; 2 European Molecular Biology Laboratory (EMBL), Gene Expression Unit, Meyerhofstrasse 1, 69117 Heidelberg, Germany; 3 Institute of Biochemistry, University of Innsbruck, Peter-Mayr-Strasse 1a, 6020 Innsbruck, Austria

## Abstract

**Background:**

The enzymes responsible for the synthesis of poly-ADP-ribose are named poly-ADP-ribose polymerases (PARP). PARP-2 is a nuclear protein, which regulates a variety of cellular functions that are mainly controlled by protein-protein interactions. A previously described non-conventional bipartite nuclear localization sequence (NLS) lies in the amino-terminal DNA binding domain of PARP-2 between amino acids 1–69; however, this targeting sequence has not been experimentally examined or validated.

**Results:**

Using a site-directed mutagenesis approach, we found that lysines 19 and 20, located within a previously described bipartite NLS, are not required for nuclear localization of PARP-2. In contrast, lysine 36, which is located within a predicted classical monopartite NLS, was required for PARP-2 nuclear localization. While wild type PARP-2 interacted with importin α3 and to a very weak extent with importin α1 and importin α5, the mutant PARP-2 (K36R) did not interact with importin α3, providing a molecular explanation why PARP-2 (K36R) is not targeted to the nucleus.

**Conclusion:**

Our results provide strong evidence that lysine 36 of PARP-2 is a critical residue for proper nuclear targeting of PARP-2 and consequently for the execution of its biological functions.

## Background

Poly-ADP-ribosylation reactions occur both in multi- and unicellular organisms and play a major role in a wide range of biological processes, such as maintenance of genomic stability, transcriptional regulation and cell death (reviewed in [1,2]). The enzyme responsible for the synthesis of poly-ADP-ribose was named poly-ADP-ribose polymerase (PARP) (reviewed in [1,2]). For a long time, PARP-1 was thought to be the only enzyme with poly-ADP-ribosylation activity in mammalian cells; however, primary cells derived from *parp-1 *knockout mice can still synthesize poly-ADP-ribose polymers after DNA damage [3]. This led to the identification of five novel poly-ADP-ribosylating enzymes, indicating that PARP-1 belongs to a family of at least six members ([4-6] and reviewed in [1,2]). PARP-2 and PARP-1 can homo- and heterodimerize and display partially redundant functions as indicated by the embryonic lethality of the *parp1-parp2*-double gene disruption ([7] and reviewed in [8]).

Mouse PARP-2 was described as a 66 kDa nuclear protein with poly-ADP-ribosylating activity [9]. The amino-terminal region of PARP-2 (aa 1–90), containing the DNA binding SAP domain, has no significant homology with any other PARP [1]. However, it is rich in basic amino acids (27% Lys or Arg), which are likely to be involved in DNA binding (reviewed in [1]). On the other hand, these basic residues could be involved in the nuclear and/or nucleolar targeting of the protein [10]. Previous studies suggested that the nuclear localization signal (NLS) of mPARP-2 is indeed located in the amino-terminal part between aa 1–69 of the protein [9,11]. Meder et al. postulated a bipartite NLS for PARP-2, but did not provide further experimental evidences to support their hypothesis [11]. Interestingly, the amino-terminal region of human and mouse PARP-2 shows higher sequence variability compared to the highly conserved carboxy-terminal catalytic region (62% identity between the amino-terminus of mPARP-2 and hPARP-2). In cells, PARP-2 has been described to regulate different processes via protein-protein interactions mediated by its amino-terminal domain (aa 1–208; reviewed in [1]).

Karyopherins, including both importins and exportins, consitute a conserved family of mobile targeting receptors that mediate the bidirectional trafficking of macromolecules across the nuclear envelope [12,13]. Most karyopherins interact directly with cargo molecules that contain nuclear import and export signals. However, importin α functions as an adaptor that links classical NLS (cNLS)-containing proteins to importin β, which, in turn, docks the ternary complex at the nuclear-pore complex (NPC). The importin α/β heterodimer is predicted to target hundreds of proteins to the NPC and facilitate their translocation across the nuclear envelope [14]. The importin α gene family has undergone considerable expansion during the course of eukaryotic evolution. Whereas the yeast *S. cerevisiae *genome encodes a single importin α, the human genome encodes six genes that fall into three phylogenetically distinct groups [15].

The nuclear targeting signal in the simian virus 40 (SV40) large T antigen was characterized more than 20 years ago [16,17]. Since then, several pathways for nucleocytoplasmic transport have been described, of which the classical nuclear import pathway is the best characterized. cNLSs are typified by either a single cluster of basic amino acids (monopartite NLS) or two clusters of basic amino acids separated by a 10–12 amino acid linker (bipartite NLS). The SV40 large T antigen (PKKKRKV) and nucleoplasmin (KRPAATKKAGQAKKKK) cNLSs are the prototypic monopartite and bipartite cNLS [18,19]. Through alanine scanning of the Myc, monopartite SV40, and artificial bipartite SV40 cNLS, Hodel and colleagues found that the binding affinity of a cNLS for importin measured *in vitro *correlated with the steady state nuclear accumulation and import rate of the corresponding cNLS cargo *in vivo *[20,21].

Here, we demonstrate that lysine 36 in the DNA binding domain (DBD) of PARP-2, which lies within a predicted cNLS motif, is required for complex formation with the importin proteins and subsequent nuclear import of PARP-2.

## Results

### Lysine 36 and/or lysine 37 of PARP-2 are required for nuclear translocation of PARP-2

**Figure 1:**
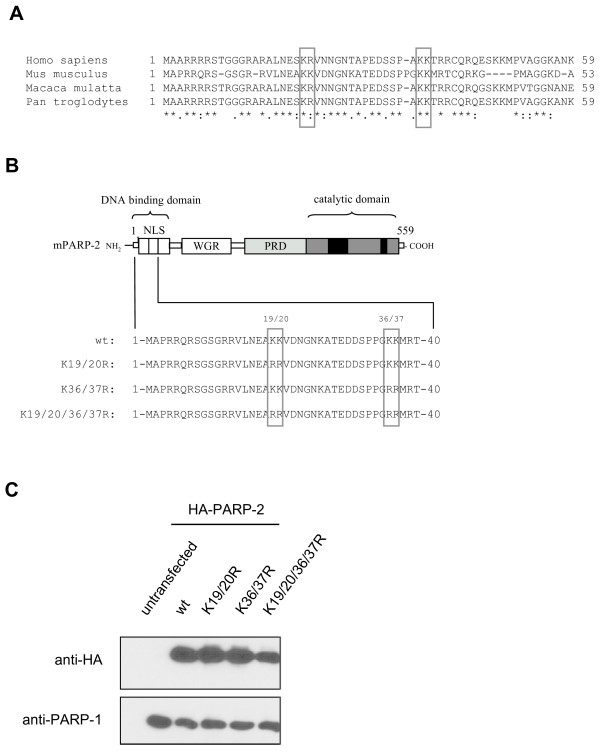
**The putative NLS of PARP-2 contains several conserved lysine residues**. **A) **Three lysines in the putative NLS of PARP-2 are conserved between different mammalian species. Sequences were obtained from NCBI and alignments were performed using ClustalW2. **B) **Schematic illustration of PARP-2 K → R mutant proteins used in this study: K19, K20, K36 and K37 were changed to arginine using site-directed mutagenesis. Double and quadruple mutants were generated. **C) **HA-tagged wild type (wt) PARP-2 or the indicated double or quadruple mutants were expressed in HEK293T cells and expression was analyzed by western blot using a monoclonal anti-HA antibody. 100 μg of whole cell extracts were used, endogenous PARP-1 levels served as loading control.

Previous experiments with GFP-fusion proteins revealed that the nuclear targeting signal of PARP-2 may be localized between aa 1–69 ([11] and Fig. 1A). This region of the protein was previously postulated to contain a bipartite cNLS; however, this sequence would be an atypical bipartite cNLS as the linker separating the two basic regions is longer than the typical 10–12 amino acid linker. This region does contain a predicted monopartite cNLS that closely matches the canonical SV40 cNLS sequence. To assess whether these sequences are important for nuclear translocation of PARP-2, mutant forms of PARP-2, K19/20R, K36/37R, and K19/20/36/37R, were generated by replacing the lysine residues with arginine residues to maintain the positive charge of the amino acids (Fig. 1B). To exclude the possibility that these amino acid changes altered the stability of the mutated PARP-2, wild type and all mutant forms were expressed as HA-tagged proteins in 293T cells and detected by immunoblot using an anti-HA antibody (Fig. 1C). Immunoblot analysis revealed that all mutants were expressed at a level comparable to wild type PARP-2.

**Figure 2:**
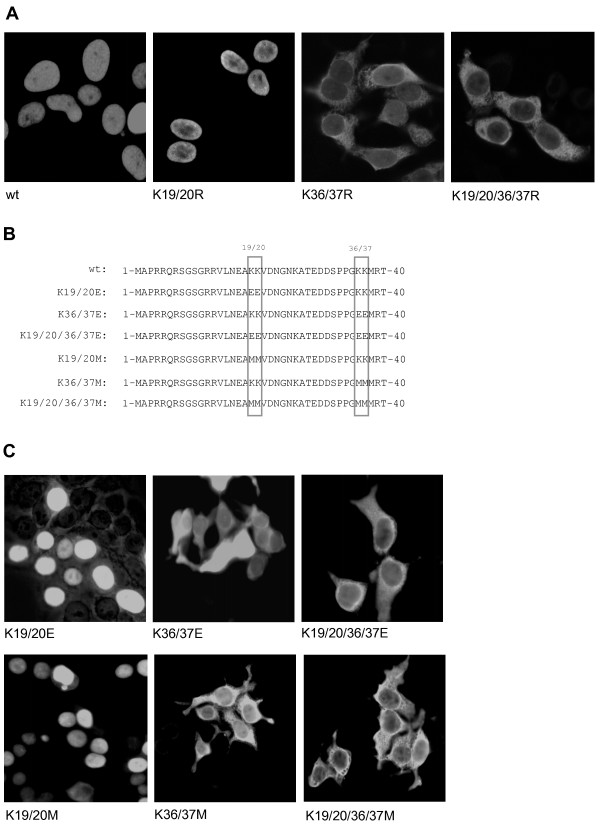
**Lysine 36 and/or lysine 37 of PARP-2 are required for nuclear localization**. **A) **HEK293T cells were transfected with HA-tagged wild type (wt) PARP-2 or with the indicated mutants. Cells were fixed with methanol for subsequent detection of HA-tagged proteins by immunofluorescence using an anti-HA antibody and a FITC-conjugated anti-mouse antibody. Representative confocal images are presented. **B) **Lysines 19, 20, 36 and 37 of PARP-2 were changed to glutamic acid or methionine as indicated. **C) **The nuclear localization is independent of the charge but dependent on the structure of the NLS. As for Fig. 2A, HEK293T cells were transfected with HA-tagged wild type (wt) PARP-2 or with the indicated K → E and K → M mutants and overexpressed proteins were detected as described for Fig. 2A. Representative confocal images are presented.

The PARP-2 mutants were transiently transfected and localization was assayed by microscopy of PARP-2 proteins. While wild type PARP-2 and the K19/20R mutant localized in the nucleus, the K19/20/36/37R and K36/37R mutants exclusively localized in the cytoplasm (Fig. 2A). To investigate whether substitution of K36 and K37 with other amino acids altered the localization of PARP-2, similar experiments were repeated with different amino acid susbstitutions. Overexpression of PARP-2 with K19/20, K36/37 or all four residues mutated to glutamate or methionine showed that K → E or K → M substitution of K36/37, but not of K19/20 altered the localization of PARP-2 to a similar extent as the K → R substitution (Fig. 2B and 2C), suggesting that K36 and/or K37 are required for the nuclear translocation of PARP-2, whereas K19 and K20 did not seem to play a role in this process.

### Leptomycin B does not change cellular localization of PARP-2 mutant K36/37R

**Figure 3:**
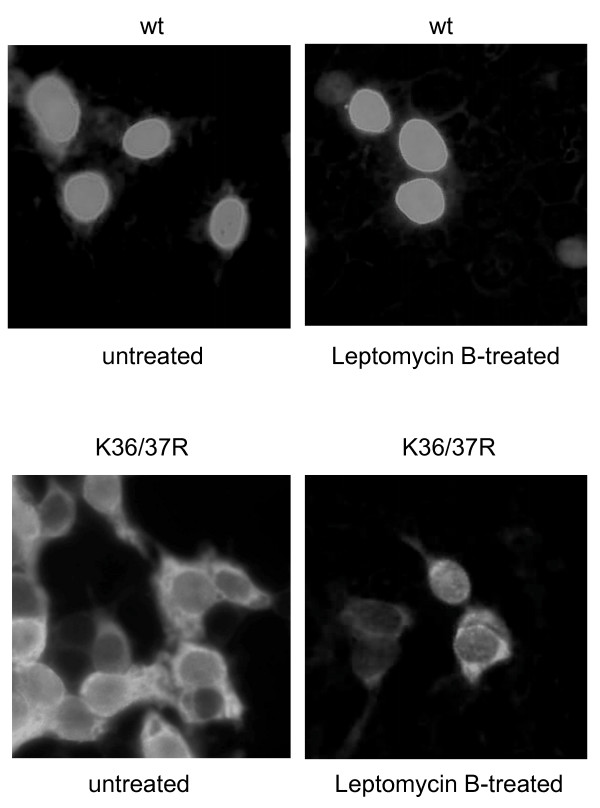
**Leptomycin B does not alter localization of the PARP-2 mutant K36/37R**. HEK293T cells were transfected with HA-tagged wild type (wt) PARP-2 (upper panel) or with the mutant K36/37R (lower panel) and treated with Leptomycin B (LMB) to inhibit nuclear export, followed by immunofluorescence as described in Fig. 2. Representative images are presented.

Next, we investigated whether the cytoplasmic localization of the mutated PARP-2 protein K36/37R is caused by an abrogated nuclear import or by an accelerated nuclear export of a transiently nuclear localized PARP-2 mutant. Cells were transfected with wild type or mutant PARP-2 and subsequently treated with Leptomycin B (LMB), a well-characterized inhibitor of CRM-1-mediated nuclear export [22-24]. Treatment with LMB did not induce any changes in the cellular localization of the PARP-2 mutant K36/37R (Fig. 3), indicating that K36 and/or K37 are more likely to impact nuclear import of PARP-2 than a classical NES-mediated export process.

### Lysine 36 but not lysine 37 of PARP-2 is required for nuclear localization of PARP-2

**Figure 4:**
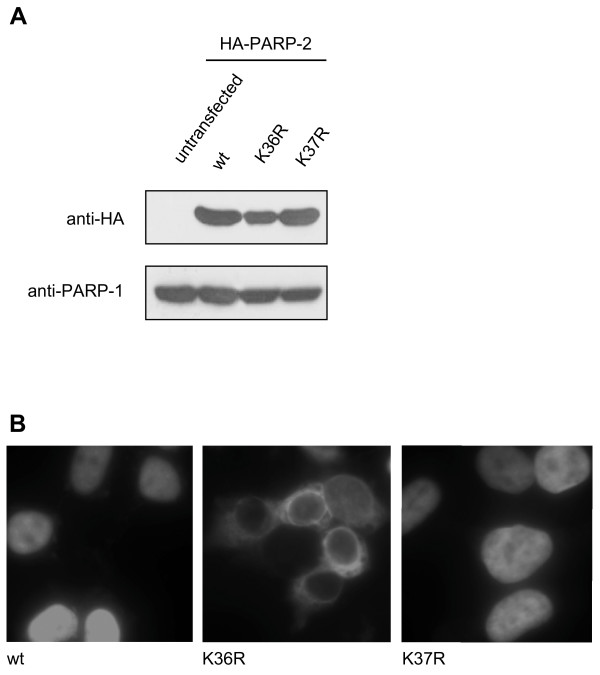
**Lysine 36 but not lysine 37 of PARP-2 is critical for nuclear localization**. **A) **HA-tagged wild type (wt) PARP-2 or the indicated single mutants were expressed in HEK293T cells and expression was analyzed by western blot using a monoclonal anti-HA antibody. 50 μg of whole cell extracts were used, endogenous PARP-1 levels served as loading control. **B) **HEK293T cells were transfected with HA-tagged wild type (wt) PARP-2 or with the PARP-2 mutants K36R and K37R. HA-tagged proteins were detected by immunofluorescence as described for Figure 2A. Representative images are presented.

In order to further investigate the requirement for K36 and K37 in the nuclear localization of PARP-2, K → R single mutants were created at each position. Interestingly, similar experiments performed with the wild type and the single mutants of PARP-2 possessing K36R and K37R substitutions revealed, that both mutants were stably expressed at levels comparable to wild type PARP-2 and that only lysine 36 was important for the nuclear accumulation of PARP-2 (Fig. 4A and 4B). In contrast to earlier reports [11], no nucleolar staining was observed under the tested conditions. These experiments identified K36 as an important residue for the nuclear localization of PARP-2 *in vivo*.

### Lysine 36 is important for binding to importin α3

**Figure 5:**
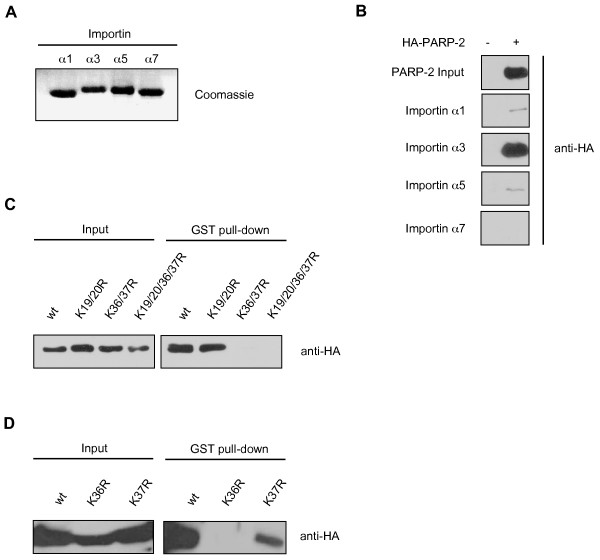
**Lysine 36 of PARP-2 is necessary for the binding of PARP-2 to importin α3**. **A) **Importins α1, α3, α5 and α7 were expressed as GST-fusion proteins in *E. coli *and purified with Glutathione Sepharose 4B beads. Expression was checked by SDS-PAGE followed by Coomassie staining. **B) **PARP-2 binds mostly to importin α3 and to a lower extent to importin α1 and α5. Purified GST-importins were incubated with whole cell extracts from HEK293T cells, either untransfected or transfected with wild type HA-PARP-2, then western blot analysis was performed using an anti-HA antibody. **C) **Lysines 36/37 are required for the binding of PARP-2 to importin α3. Purified GST-importin α3 was incubated with whole cell extracts from HEK293T cells transfected with either wild type (wt) HA-PARP-2 or with the indicated double and quadruple mutants. Proteins were separated by SDS-PAGE and analyzed by western blot using an anti-HA antibody. **D) **Lysine 36 but not lysine 37 is required for the binding of PARP-2 to importin α3. GST-importin α3 was bound to Glutathione Sepharose 4B and incubated with whole cell extracts from HEK293T cells expressing either wild type (wt) PARP-2 or the indicated single mutants. PARP-2 bound to importin α3 was detected using an anti-HA antibody.

One possibility to confirm the functional cNLS targeting sequence is to perform interaction studies with the classical NLS import receptor, importin α. In order to test whether PARP-2 interacts with importin α, we performed GST pull-down experiments with different recombinant purified GST-fusion proteins of human importin α (α1, α3, α5 and α7; Fig. 5A) and cell extracts containing overexpressed wild type or different mutated PARP-2 proteins. PARP-2 was detected in the bound fraction following the pull-down assay by western blot analysis. Wild type PARP-2 formed a complex with importin α3 and to a very weak extent also with importin α1 and importin α5, but not with importin α7 (Fig. 5B). Experiments with purified wild type and mutated PARP-2 (K36/37R, K36R and K37R) revealed that the mutant proteins K36/37R and K36R did not bind importin α3, while the K37R mutant did bind, suggesting that K36 is a critical residue of PARP-2 essential for its interaction with importin α3 and its nuclear translocation (Fig. 5C and 5D).

## Discussion

PARP-2 regulates different cellular functions. Here, we provide both biochemical and functional evidence that substitution of lysine residue 36 efficiently inhibits localization of PARP-2 to the nucleus. Functional analyses revealed that lysine 36 is important for complex formation with importin α3.

Lysine residues are central components of classical NLS motifs (reviewed by [25]) as their positive charge mediates the interaction with importin receptors [26]. Here we provide evidence that K36 of PARP-2 is an important residue required for the nuclear translocation of PARP-2 and for complex formation with importin α3, as mutation of this residue was sufficient to disrupt association with the import machinery and subsequently alter PARP-2 nuclear localization. Interestingly, lysine 36 is conserved between mouse and human PARP-2, suggesting that the described findings might also apply for the human counterpart. Together, our data indicate that the nuclear import of human and murine PARP-2 is mediated by a conserved classical monopartite NLS but not through a bipartite NLS as previously proposed [11].

The formation of the importin-α/β-cNLS cargo ternary complex is the first step in the nuclear transport of hundreds of different nuclear proteins, and, as such, is tightly regulated [15]. The relationship of importin α/β with its cNLS cargo is by necessity bipolar, because it forms highly selective and tight complexes in the cytoplasm and then switches to an extremely low affinity state in the nucleus to release the cargo. When importin α is not bound to importin β, an autoinhibitory sequence within the amino-terminal domain apparently interacts with the NLS-binding pocket [27]. This interaction is not exceptionally strong because cNLS cargos can still bind to importin α in the absence of importin β, albeit with significantly lower affinity. The order of importin α binding to cNLS cargo and importin β is not known. The observed lack of importin α3 binding by the PARP-2 mutant (K36R) clearly indicates that this lysine is required for the interaction with importin α and subsequently for nuclear translocation.

Recently, it has become evident that importin α receptors have independent roles in the assembly of macromolecular structures. Genetic analyses of yeast importin α mutants identified several alleles that confer defects in chromosome and nuclear segregation, altered mitotic spindle structure and deficits in the ubiquitin-mediated protein degradation pathway [28-31]. Mechanistic studies on the roles of importin αs in mitosis, spindle assembly and nuclear envelope biogenesis point more directly to activities which are independent of the housekeeping roles of importin α in nuclear transport. The observed interaction of PARP-2 with importin α might thus not only be important for its nuclear translocation but might have an additional physiological function in maintaining the integrity of the genome. Inactivation of the *parp-2 *gene in mice revealed that PARP-2 may be involved in the surveillance and maintenance of genome integrity, indicated by the sensitivity of these mice to ionizing radiation [7].

Others have reported that PARP-2 is enriched within the whole nucleolus and partially colocalizes with the nucleolar factor nucleophosmin/B23 [11]. Using partial cDNA fragments in-frame with the carboxy-terminus of EGFP the authors described a putative nuclear localization signal and a nucleolar localization signal within the amino-terminal domain of PARP-2 (aa 1–69). Our studies revealed that overexpressed PARP-2 was only found equally distributed in the nucleus, but in contradiction to this previous report, was never observed in the nucleolus of the cell. This discrepancy could be explained by the different experimental approaches used. Meder et al. studied the nucleolar localization of PARP-2 with GFP-fusion proteins, while our studies were performed with non-GFP tagged full-length proteins. Remarkably, PARP-1 nucleolar accumulation was not observed when endogenous or overexpressed PARP-1 localization was analyzed by a conventional immunofluorecence protocol as described in Methods using specific anti-PARP-1 antibodies (data not shown). Only applying the fixation protocol described in Meder et al. [11], which led to the decomposition of the cell and loss of cytoplasm, revealed the reported nucleolar staining of PARP-1, suggesting that the fixation protocol influences the nucleolar localization of proteins or the detection of proteins within the nucleolus.

Recently, acetylation of lysine residues by histone acetyltransferases (HATs), such as p300/CBP (CREB-binding protein) and PCAF (p300/CBP-associated factor), has been proposed as a new mechanism for modulating cellular localization [32-36]. HATs trigger the transfer of an acetyl group from acetyl coenzyme A to the epsilon-amino group of a lysine residue not only on core histones but also on about 40 transcription factors and on more than 30 other proteins [37]. We recently published that both lysines 36 and 37 of PARP-2 are indeed acetylated *in vitro *and *in vivo *and that acetylation influences both DNA binding and auto-ADP-ribosylation of PARP-2 [38].

## Conclusion

Taken together, our results provide evidence that PARP-2 accumulates in the nucleus and that lysine 36, which is located within a monopartite cNLS, is important for binding of PARP-2 to importin α3 and for the nuclear translocation of PARP-2.

## Methods

### Plasmids

Mammalian expression vectors for wild type PARP-2 and all mutants used in this study were obtained by cloning the corresponding PCR products into pphCMV-HA. PARP-2 mutants were generated by a site directed mutagenesis procedure and confirmed by sequencing. Bacterial expression vectors for human GST-importins α1, α3, α5 and α7 were provided by Dr. Riku Fagerlund (Departments of Viral Diseases and Immunology and Epidemiology and Health Promotion, National Public Health Institute, FIN-00300, Helsinki, Finland, [39]).

### Expression and purification of recombinant proteins

GST-tagged importins were expressed in *E. coli *strain BL21-D3-Gold. All purified proteins were analyzed by Coomassie staining and confirmed by western blot analysis using the corresponding antibodies.

### Cell culture and transient transfections, treatment with LMB and immunofluorescence

HEK293T cells were grown in Hepes-buffered DMEM-Glutamax-I (Invitrogen) containing 4.5 g/L glucose and 10% FCS US/certified (Invitrogen) and supplemented with 50 U/ml penicillin, 50 μg/ml streptomycin (Invitrogen) and MEM non-essential amino acids (MEM NEAA, Invitrogen). Cells were transfected using calcium phosphate procedures as described in [40]. For the experiments with Leptomycin B (LMB), cells were treated with a final concentration of 20 ng/ml LMB for 4–16 hrs. For detection of overexpressed proteins by immunofluorescence, HEK293T cells were fixed for 10 minutes in ice-cold 100% methanol in the absence of detergents and unspecific binding sites were blocked with 2% BSA/0.1% Triton X-100 prior to staining with primary and FITC-conjugated secondary antibodies in the presence of 2% BSA/0.1% Triton X-100 according to the manufacturer's protocol (Covance) using confocal (Leica SP2, 40× oil-immersion, NA 1.25, zoom-in) or standard fluorescence microscopy (Olympus Mx51, 100× oil-immersion, NA 1.3).

### Western blot analysis and antibodies

Western blot analyses were performed as described previously [41]. Anti-myc-9E10 (sc-2027) antibodies were obtained from Santa Cruz Biotechnology, anti-HA (MMS-101P) was obtained from COVANCE. Antibodies against mouse PARP-1 and PARP-2 were generated in house (the generation of antibodies against mouse PARP-1 has been described previously [42,43]).

### *In vitro *interaction and GST pull-down assays

Purified recombinant proteins fused to GST were bound to Glutathione Sepharose 4B according to the manufacturer's protocols (Amersham Biosciences). GST pull-down assays were performed as described previously [41,42]. GST pull-down-buffers contain: 50 mM Tris [pH 8.0], 150 mM NaCl, 0.5% NP-40, 0.5 mM DTT, 1 mM PMSF, 100 μM bestatin, 3 μM pepstatin A, 5 μM leupeptin. Bound proteins were dissolved by SDS PAGE and subsequently analyzed by western blot.

## Authors' contributions

SSH carried out the molecular studies and drafted the manuscript. MA carried out the molecular studies with the single PARP-2 mutants and drafted the figures and helped to draft the manuscript. POH participated in the design of the study, carried out the site directed mutagenesis and helped to draft the mansucript. TV participated in the design of the study. MF purified recombinant proteins. MOH conceived the study and participated in its design and coordination and helped to draft the manuscript. All authors read and approved the final manuscript.

## References

[B1] Hassa PO, Hottiger MO (2008). The diverse biological roles of mammalian PARPS, a small but powerful family of poly-ADP-ribose polymerases. Front Biosci.

[B2] Hassa PO, Haenni SS, Elser M, Hottiger MO (2006). Nuclear ADP-ribosylation reactions in mammalian cells: where are we today and where are we going?. Microbiol Mol Biol Rev.

[B3] Shieh WM, Ame JC, Wilson MV, Wang ZQ, Koh DW, Jacobson MK, Jacobson EL (1998). Poly(ADP-ribose) polymerase null mouse cells synthesize ADP-ribose polymers. J Biol Chem.

[B4] Marsischky GT, Wilson BA, Collier RJ (1995). Role of glutamic acid 988 of human poly-ADP-ribose polymerase in polymer formation. Evidence for active site similarities to the ADP-ribosylating toxins. J Biol Chem.

[B5] Rolli V, O'Farrell M, Menissier-de Murcia J, de Murcia G (1997). Random mutagenesis of the poly(ADP-ribose) polymerase catalytic domain reveals amino acids involved in polymer branching. Biochemistry.

[B6] Ruf A, Rolli V, de Murcia G, Schulz GE (1998). The mechanism of the elongation and branching reaction of poly(ADP-ribose) polymerase as derived from crystal structures and mutagenesis. J Mol Biol.

[B7] Menissier de Murcia J, Ricoul M, Tartier L, Niedergang C, Huber A, Dantzer F, Schreiber V, Ame JC, Dierich A, LeMeur M, Sabatier L, Chambon P, de Murcia G (2003). Functional interaction between PARP-1 and PARP-2 in chromosome stability and embryonic development in mouse. Embo J.

[B8] Schreiber V, Dantzer F, Ame JC, de Murcia G (2006). Poly(ADP-ribose): novel functions for an old molecule. Nat Rev Mol Cell Biol.

[B9] Ame JC, Rolli V, Schreiber V, Niedergang C, Apiou F, Decker P, Muller S, Hoger T, Menissier-de Murcia J, de Murcia G (1999). PARP-2, A novel mammalian DNA damage-dependent poly(ADP-ribose) polymerase. J Biol Chem.

[B10] Dang CV, Lee WM (1989). Nuclear and nucleolar targeting sequences of c-erb-A, c-myb, N-myc, p53, HSP70, and HIV tat proteins. J Biol Chem.

[B11] Meder VS, Boeglin M, de Murcia G, Schreiber V (2005). PARP-1 and PARP-2 interact with nucleophosmin/B23 and accumulate in transcriptionally active nucleoli. J Cell Sci.

[B12] Fried H, Kutay U (2003). Nucleocytoplasmic transport: taking an inventory. Cell Mol Life Sci.

[B13] Macara IG (2001). Transport into and out of the nucleus. Microbiol Mol Biol Rev.

[B14] Lange A, Mills RE, Lange CJ, Stewart M, Devine SE, Corbett AH (2007). Classical nuclear localization signals: definition, function, and interaction with importin alpha. J Biol Chem.

[B15] Goldfarb DS, Corbett AH, Mason DA, Harreman MT, Adam SA (2004). Importin alpha: a multipurpose nuclear-transport receptor. Trends Cell Biol.

[B16] Kalderon D, Richardson WD, Markham AF, Smith AE (1984). Sequence requirements for nuclear location of simian virus 40 large-T antigen. Nature.

[B17] Kalderon D, Roberts BL, Richardson WD, Smith AE (1984). A short amino acid sequence able to specify nuclear location. Cell.

[B18] Robbins J, Dilworth SM, Laskey RA, Dingwall C (1991). Two interdependent basic domains in nucleoplasmin nuclear targeting sequence: identification of a class of bipartite nuclear targeting sequence. Cell.

[B19] Dingwall C, Laskey RA (1991). Nuclear targeting sequences – a consensus?. Trends Biochem Sci.

[B20] Hodel MR, Corbett AH, Hodel AE (2001). Dissection of a nuclear localization signal. J Biol Chem.

[B21] Hodel AE, Harreman MT, Pulliam KF, Harben ME, Holmes JS, Hodel MR, Berland KM, Corbett AH (2006). Nuclear localization signal receptor affinity correlates with *in vivo* localization in *Saccharomyces cerevisiae*. J Biol Chem.

[B22] Fornerod M, Ohno M, Yoshida M, Mattaj IW (1997). CRM1 is an export receptor for leucine-rich nuclear export signals. Cell.

[B23] Fukuda M, Asano S, Nakamura T, Adachi M, Yoshida M, Yanagida M, Nishida E (1997). CRM1 is responsible for intracellular transport mediated by the nuclear export signal. Nature.

[B24] Wolff B, Sanglier JJ, Wang Y (1997). Leptomycin B is an inhibitor of nuclear export: inhibition of nucleo-cytoplasmic translocation of the human immunodeficiency virus type 1 (HIV-1) Rev protein and Rev-dependent mRNA. Chem Biol.

[B25] Poon IK, Jans DA (2005). Regulation of nuclear transport: central role in development and transformation?. Traffic.

[B26] Conti E, Uy M, Leighton L, Blobel G, Kuriyan J (1998). Crystallographic analysis of the recognition of a nuclear localization signal by the nuclear import factor karyopherin alpha. Cell.

[B27] Kobe B (1999). Autoinhibition by an internal nuclear localization signal revealed by the crystal structure of mammalian importin alpha. Nat Struct Biol.

[B28] Loeb JD, Schlenstedt G, Pellman D, Kornitzer D, Silver PA, Fink GR (1995). The yeast nuclear import receptor is required for mitosis. Proc Natl Acad Sci USA.

[B29] Kussel P, Frasch M (1995). Yeast Srp1, a nuclear protein related to Drosophila and mouse pendulin, is required for normal migration, division, and integrity of nuclei during mitosis. Mol Gen Genet.

[B30] Tabb MM, Tongaonkar P, Vu L, Nomura M (2000). Evidence for separable functions of Srp1p, the yeast homolog of importin alpha (Karyopherin alpha): role for Srp1p and Sts1p in protein degradation. Mol Cell Biol.

[B31] Yano R, Oakes ML, Tabb MM, Nomura M (1994). Yeast Srp1p has homology to armadillo/plakoglobin/beta-catenin and participates in apparently multiple nuclear functions including the maintenance of the nucleolar structure. Proc Natl Acad Sci USA.

[B32] Bannister AJ, Miska EA, Gorlich D, Kouzarides T (2000). Acetylation of importin-alpha nuclear import factors by CBP/p300. Curr Biol.

[B33] Soutoglou E, Katrakili N, Talianidis I (2000). Acetylation regulates transcription factor activity at multiple levels. Mol Cell.

[B34] Spilianakis C, Papamatheakis J, Kretsovali A (2000). Acetylation by PCAF enhances CIITA nuclear accumulation and transactivation of major histocompatibility complex class II genes. Mol Cell Biol.

[B35] Bonaldi T, Talamo F, Scaffidi P, Ferrera D, Porto A, Bachi A, Rubartelli A, Agresti A, Bianchi ME (2003). Monocytic cells hyperacetylate chromatin protein HMGB1 to redirect it towards secretion. Embo J.

[B36] Santos-Rosa H, Valls E, Kouzarides T, Martinez-Balbas M (2003). Mechanisms of P/CAF auto-acetylation. Nucleic Acids Res.

[B37] Yang XJ (2004). The diverse superfamily of lysine acetyltransferases and their roles in leukemia and other diseases. Nucleic Acids Res.

[B38] Haenni SS, Hassa PO, Altmeyer M, Fey M, Imhof R, Hottiger MO (2008). Identification of lysines 36 and 37 of PARP-2 as targets for acetylation and auto-ADP-ribosylation. Int J Biochem Cell Biol.

[B39] Melen K, Fagerlund R, Franke J, Kohler M, Kinnunen L, Julkunen I (2003). Importin alpha nuclear localization signal binding sites for STAT1, STAT2, and influenza A virus nucleoprotein. J Biol Chem.

[B40] Perkins ND, Felzien LK, Betts JC, Leung K, Beach DH, Nabel GJ (1997). Regulation of NF-kappaB by cyclin-dependent kinases associated with the p300 coactivator. Science.

[B41] Hassa PO, Buerki C, Lombardi C, Imhof R, Hottiger MO (2003). Transcriptional coactivation of nuclear factor-kappaB-dependent gene expression by p300 is regulated by poly(ADP)-ribose polymerase-1. J Biol Chem.

[B42] Hassa PO, Haenni SS, Buerki C, Meier NI, Lane WS, Owen H, Gersbach M, Imhof R, Hottiger MO (2005). Acetylation of poly(ADP-ribose) polymerase-1 by p300/CREB-binding protein regulates coactivation of NF-kappaB-dependent transcription. J Biol Chem.

[B43] Petrilli V, Herceg Z, Hassa PO, Patel NS, Di Paola R, Cortes U, Dugo L, Filipe HM, Thiemermann C, Hottiger MO, Cuzzocrea S, Wang ZQ (2004). Noncleavable poly(ADP-ribose) polymerase-1 regulates the inflammation response in mice. J Clin Invest.

